# Autism spectrum traits predict the neural response to eye gaze in typical individuals

**DOI:** 10.1016/j.neuroimage.2011.10.075

**Published:** 2012-02-15

**Authors:** Lauri Nummenmaa, Andrew D. Engell, Elisabeth von dem Hagen, Richard N.A. Henson, Andrew J. Calder

**Affiliations:** a Brain Research Unit, Low Temperature Laboratory and Department of Biomedical Engineering and Computational Science, Aalto University School of Science, Finland; b Turku PET Centre, Finland; c MRC Cognition and Brain Sciences Unit, Cambridge, UK; d Human Neuroscience Laboratory, Psychology Department, Yale University, New Haven, CT, USA

**Keywords:** Eye gaze, fMRI, Autism spectrum, Attention, Face perception

## Abstract

Autism Spectrum Disorders (ASD) are neurodevelopmental disorders characterised by impaired social interaction and communication, restricted interests and repetitive behaviours. The severity of these characteristics are posited to lie on a continuum extending into the typical population, and typical adults' performance on behavioural tasks that are impaired in ASD is correlated with the extent to which they display autistic traits (as measured by Autism Spectrum Quotient, AQ). Individuals with ASD also show structural and functional differences in brain regions involved in social perception. Here we show that variation in AQ in typically developing individuals is associated with altered brain activity in the neural circuit for social attention perception while viewing others' eye gaze. In an fMRI experiment, participants viewed faces looking at variable or constant directions. In control conditions, only the eye region was presented or the heads were shown with eyes closed but oriented at variable or constant directions. The response to faces with variable vs. constant eye gaze direction was associated with AQ scores in a number of regions (posterior superior temporal sulcus, intraparietal sulcus, temporoparietal junction, amygdala, and MT/V5) of the brain network for social attention perception. No such effect was observed for heads with eyes closed or when only the eyes were presented. The results demonstrate a relationship between neurophysiology and autism spectrum traits in the typical (non-ASD) population and suggest that changes in the functioning of the neural circuit for social attention perception is associated with an extended autism spectrum in the typical population.

## Highlights

► Autistic spectrum might extend to typically developing (TD) individuals. ► We studied TD individuals with varying Autism Spectrum Quotient (AQ). ► AQ correlated with BOLD response to viewing variable vs. constant eye gaze. ► AQ did not correlate with response to directional control stimuli. ► Neurophysiology and autism spectrum traits are associated in non-AS individuals.

## Introduction

Autism spectrum disorders (ASD) are characterized by abnormal social interaction and communication, severely restricted interests and repetitive behaviour. ASD have a range of clinical phenotypes from mild to severe, however an even wider continuum of social-communicative ability has been proposed extending into the general or typical population (Baron-Cohen, 1995; Baron-Cohen et al., 2001b; Frith, 1991). Initial support comes from studies demonstrating that the degree of autistic traits measured by Autism Spectrum Quotient (AQ; Baron-Cohen et al., 2001b), in both ASD and typical populations, is related to performance on behavioural tasks that show impairments in ASD, including self-focussed attention (Lombardo et al., 2007), the ability to draw mentalistic inferences from the eyes (Baron-Cohen et al., 2001a), and attentional cueing from eye gaze (Bayliss and Tipper, 2005; Bayliss and Tipper, 2006). However, stronger evidence for a continuum extending into the typical population would involve a demonstration that the neural response to these sorts of social tasks or stimuli is related to typical participants' scores on the AQ.

Neuroimaging has shown structural and functional impairments in ASD in ‘social’ brain regions involved in processing goal-directed actions and biological motion (superior temporal sulcus, STS), theory of mind (medial prefrontal cortex; mPFC and temporo-parietal junction; TPJ), and emotion (amygdala) (see reviews in Baron-Cohen et al., 2000; Di Martino et al., 2009; Frith, 2001; Zilbovicius et al., 2006). A number of these areas, together with components of the attention system, are recruited during gaze perception (Nummenmaa and Calder, 2009), and we will refer to them as the ‘social attention’ network. Since individuals with ASD also show an abnormal neural response to gaze cues (Grice et al., 2005; Pelphrey et al., 2005; Senju et al., 2005), gaze perception provides a well-grounded model for studying neurophysiological correlates of the autistic spectrum in the general population. Our recent work in typical participants showing that individual differences in AQ predict changes in the structure and function of the posterior STS (pSTS), a central component of the social attention network, provides further reason to predict that a relationship between AQ and the social attention network might be found (von dem Hagen et al., 2011). Specifically, this study found a reduction in white matter in pSTS of individuals with higher AQ scores. This was accompanied by an increased task-independent deactivation in high-AQ subjects, akin to ‘resting state’ activity, in the same area of pSTS. Here we investigate whether the BOLD response in the pSTS region, and wider components of the social attention network with which it is connected (Nummenmaa et al., 2010), show a significant relationship with AQ in response to viewing gaze stimuli. This would provide the first direct evidence that individual variation in autism spectrum traits in typical participants impact on the neural correlates of social processing. We addressed this in the context of a functional magnetic resonance imaging (fMRI) study.

**Fig. 1:**
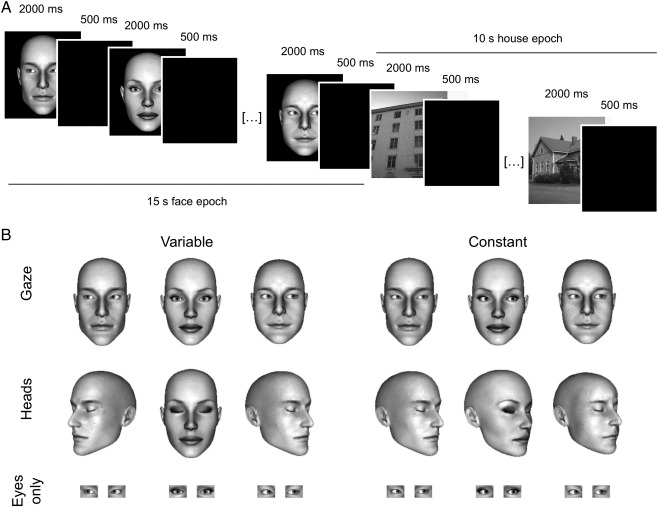
(A) Experimental design. Participants viewed 15 s epochs of faces (6 presentations) intermixed with 10 s epochs of houses (4 presentations). Identity always changed between two consecutive face/house presentations. (B) Illustration of stimuli. Participants viewed gaze (top row), head (heads with eyes closed, middle row) or eyes-only (eyes with face masked, bottom row) stimuli that were either variable (left panel) or constant (right panel) with respect to direction within each block.

Participants viewed epochs of faces gazing in ‘variable’ directions (i.e., left, direct, and right) or a constant direction (e.g., all left), implying rapid changes in their focus of attention or interest, or no change, respectively (Fig. 1). A second, control condition comprised ‘variable’ and ‘constant’ epochs of oriented heads with eyes closed, thus physical direction was again variable or constant but without concomitant changes in the faces' focus of attention and interest. ASD is known to impact primarily on the ability to draw mental inferences from social attention cues (e.g., that a person is *interested* in something to their left) rather than discrimination of their perceived physical direction (Baron-Cohen et al., 1995; Nation and Penny, 2008). Consequently, an effect of AQ on the social attention network was predicted to be most apparent for a comparison of the variable versus constant gaze conditions because mental inferences regarding the faces' should be greater for the rapidly changing foci of interest conveyed by the variable gaze condition relative to the constant gaze condition in which the focus of interest remained fixed. In contrast, we predicted that an effect of AQ should be absent for a comparison of the variable versus constant head conditions because heads with eyes closed convey directional information only.

Our study also included a third ‘Eyes only’ condition in which gaze was again variable or constant but only the eyes were visible. For this condition we predicted a reduced or absent relationship with AQ for a comparison of the variable and constant conditions since previous research has shown that perception of gaze direction, or gaze following, is heavily influenced by the orientation of the head. For example, significant gaze cueing to the left (or right) has been reported using gaze cues in which the heads are rotated to the left (or right) and the gaze directed towards the observer (Hietanen, 1999). Moreover, these effects were significantly larger than those observed using leftward (or rightward) oriented heads with their gaze oriented in the same direction as the heads. This suggests that the cueing effect is not dependent on the direction of the gaze alone, but is contingent on directional cues conveyed by the head *and* eyes.

## Materials and methods

### Participants

Eighteen right-handed typical, healthy volunteers (4 males; aged 18 to 30 years; mean age = 24 years) with normal or corrected to normal vision participated in the study in return for payment. Individuals with a history of neurological or psychiatric disease or currently taking medication affecting the central nervous system were excluded. All provided written informed consent as a part of a protocol approved by the Suffolk Research Ethics Committee. Participants completed the AQ before scanning. This questionnaire contains 50 questions measuring the extent of autism spectrum characteristics, and has good test–retest reliability and internal consistency (Baron-Cohen et al., 2001b).

### Design and procedure

Stimuli and design are summarized in Fig. 1. The stimuli were prepared from 10 computer-generated faces (5 males and 5 females). Participants were shown three types of facial stimuli. A ‘Gaze’ condition comprised full-face views of faces with eyes oriented 25° left (L), 0° (direct), or 25° right (R). In a second ‘Heads’ condition, heads with eyes closed were oriented 25°L, 0°, or 25°R. In a third, ‘Eyes only’ condition, the gaze was again oriented 25°L, 0°, or 25°R, however, the majority of the head was masked leaving only the eyes visible. The facial stimuli were presented in 15 s epochs containing six presentations of one of the three facial stimulus types. The epochs were divided into two further conditions — ‘variable’ and ‘constant’. For example, in the ‘constant’ condition for the Gaze stimuli, all faces displayed the same gaze direction (i.e. all 25°L, all 0° or all 25°R), whereas the variable condition comprised continually changing gaze directions (i.e. 25°L, 0° and 25°R in random order). Similarly, the variable and constant conditions for the Heads and Eyes-only stimuli showed different or repeated head and gaze orientations.

Each facial stimulus was presented for 2 s, followed by a 500 ms blank screen to prevent apparent motion. Consecutive faces never showed the same identity. The task was to categorize the gender of each face. Seven epochs of each category were presented. The order of epochs was fixed for each participant (e.g. gaze, heads, eyes-only, gaze …) counterbalanced across participants. The face epochs were interleaved with 10 s epochs of house images. This helped to reduce activation in the gaze network between face conditions and acted as a baseline condition. Note that houses were used in preference to a fixation (rest) condition because activation at rest varies between individuals with Autism and typical controls in brain areas associated with social processing, and is correlated with AQ in typical participants (Kennedy and Courchesne, 2008; Kennedy et al., 2006; von dem Hagen et al., 2011). To reduce task switching, participants were also asked to categorize the gender (‘masculine’ vs. ‘feminine’) of the house stimuli. Pilot testing showed that participants were readily able to categorise houses with these labels. The total task duration was 33 min and 50 s. The stimuli were presented via an angled mirror above the participants' eyes. The mirror reflected images back-projected onto a translucent screen in the bore of the magnet behind the participants' head. All participants practised the task outside the scanner prior to the experiment.

### Image acquisition and preprocessing

MR imaging was performed with a 3-Tesla Tim Trio Magnetic Resonance Imaging scanner (Siemens, Germany) with a head coil gradient set at the MRC Cognition and Brain Sciences Unit. Whole-brain data were acquired with an echo-planar T2*-weighted (EPI) imaging sequence, sensitive to the blood–oxygen-level-dependent (BOLD) signal contrast (40 oblique slices, 3 mm slice thickness; TR = 2424 ms; TE = 30 ms; flip angle = 78°; FOV 192 mm; voxel size = 3 × 3 × 3 mm). The first 3 volumes were discarded to allow for equilibration effects. T1-weighted structural images were acquired at a resolution of 1 × 1 × 1 mm. Data were preprocessed and analyzed using SPM5 software (www.fil.ion.ucl.ac.uk/spm/). The EPI images were sinc interpolated in time to correct for slice time differences and realigned to the first scan by rigid body transformations to correct for head movements. EPI and structural images were coregistered and normalized to the T1 standard template in MNI space (Montreal Neurological Institute (MNI) — International Consortium for Brain mapping) using linear and non-linear transformations, and smoothed with a Gaussian kernel of FWHM 8-mm.

### Analysis of regional effects

A random effects model was implemented using a two-stage process, of within (first level) and between (second level) subjects modelling, in turn. This random-effects analysis assessed effects on the basis of inter-subject variance and thus allowed inferences about the population that the participants were drawn from. For each participant we used a GLM to assess regionally specific effects of task parameters on BOLD indices of activation. The model included two experimental factors — stimulus type (Gaze, Heads, and Eyes-only) and presentation format (variable vs. constant); effects of no interest (realignment parameters) were also included to account for motion-related variance. Low-frequency signal drift was removed using a high-pass filter (cutoff 128 s) and AR(1) modelling of temporal autocorrelations was applied.

The individual contrast images were generated using the following contrasts: i) variable versus constant gaze, ii) variable vs. constant heads, and iii) variable vs. constant eyes-only. The second level analyses used these contrast images in new GLMs with AQ as a regressor (plus a constant term), from which we generated statistical images, i.e. SPM-t maps, of voxels showing positive or negative relationships with AQ. With balanced designs at the first level (i.e. similar events for each subject, in similar numbers) this second level analysis closely approximates a true mixed effects design, with both within and between subject variance. A recent meta-analysis identified that perception of others' gaze engages a network of regions comprising the pSTS/TPJ, fronto-parietal attention system, amygdala, and dorsal medial prefrontal cortex (Nummenmaa and Calder, 2009). The relationship between AQ and activation in this network was assessed with a threshold of p < 0.05 whole-brain FDR correction and minimum cluster size of 20 voxels.

## Results

### Behavioural data

The distribution of the AQ scores was as follows: range = 7–33, *M* = 16.80, *SD* = 7.60. The mean accuracy for gender classification of faces was high (86%) and exceeded chance level (50%) for all task conditions, *p*s < 0.05. However, accuracy varied slightly across task conditions, *F*(2,34) = 5.50, *p* < 0.01, η_p_^2^ = 0.24 (*M*_gaze_ = 91%, *M*_eyes-only_ = 81%, *M*_heads_ = 88%). As would be expected, accuracy for eyes-only condition was lower than that for the gaze (*p* < 0.01) or head (*p* < 0.01) conditions, for which the full face was visible; the gaze and head conditions did not significantly differ (*p* > 0.05). Median latencies for correct responses were also influenced by condition, *F*(2,34) = 34.48, *p* < 0.001, η_p_^2^ = 0.67 (*M*_gaze_ = 612 ms, *M*_eyes-only_ = 774 ms, *M*_heads_ = 613 ms). Latency for eyes-only condition was longer than that for the gaze (*p* < 0.01) or head (*p* < 0.01) conditions, with no differences between the latter two conditions (*p* > 0.05). Neither the accuracy nor the response latency correlated with the AQ scores, *r*s < 0.3, *p*s > 0.4. Accuracy and RTs were not analyzed for the house trials because responses could not be categorised as correct and incorrect.

### fMRI data

**Fig. 2:**
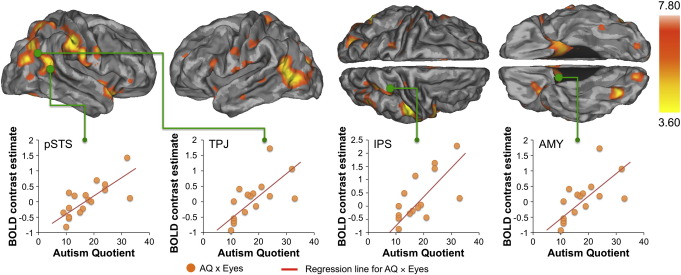
Brain regions showing positive correlations between AQ and activation to variable versus constant eye gaze direction (*p* < 0.05, whole brain FDR corrected). Colour bar denotes *t*-statistic range. The scatterplots show the association of AQ and BOLD contrast in selected regions. pSTS = posterior superior temporal sulcus, TPJ = temporo-parietal junction, IPS = intraparietal sulcus and AMY = amygdala. Scatterplots are for visualization only.

The second-level contrasts for variable vs. constant gaze, variable vs. constant heads and variable vs. constant eyes-only (or vice versa) did not yield any significant effects. Consistent with our hypothesis, however, the variable vs. constant gaze comparison yielded significant positive correlations with the AQ (Fig. 2) in a number of regions of the gaze perception network, including right pSTS (50, − 50, 18, *T* = 4.38), intraparietal sulcus (IPS) (34, − 48, 60, *T* = 4.43), bilateral amygdala (left: − 26, − 8, − 12, *T* = 4.37; right: 26, − 4, − 14, *T* = 3.81) and right TPJ (52, − 58, 8, *T* = 4.38). Additional cortical clusters were observed in the vicinity of the MT/V5 (− 42, − 66, 2, *T* = 7.87) and inferior parietal lobule (IPL) and supramarginal gyrus (SMG) (− 42, − 30, 36, *T* = 4.35). Other regions that survived our a priori threshold are summarised in Table 1.

Following the request of one of the reviewers, a secondary ROI analysis (see Supplementary Fig. 1) using independently defined ROIs confirmed that AQ correlated significantly with BOLD response to gaze specifically in regions that prior studies have linked with gaze and face perception (OFA, FFA, pSTS, IPS and amygdala) as well as mentalizing (TPJ), *p* < 0.05 FWE small-volume corrected. In contrast to the gaze condition, AQ scores did not significantly correlate with activation to the variable vs. constant heads and variable vs. constant eyes-only conditions in any brain region, even at reduced threshold (*p* < 0.05, uncorrected).

As the AQ can be split up into five subscales measuring different domains (social skills, attention switching, attention to detail, communication and imagination), we tested whether the scores for these domains would also show correlations with the variable versus constant gaze/heads/eyes only contrasts. The overall pattern was similar to that observed with the composite AQ score. Once again, the scales only correlated with the response to variable versus constant gaze, and not with variable versus constant heads or eyes-only. The correlations were also smaller in magnitude on average than those for the composite score. This may be because the range of the subscale scores is truncated in comparison with the composite AQ score. Subscales for attention to detail, imagination and social skills showed the largest correlations with the variable versus constant gaze contrast, while attention switching and communication showed the smallest correlations (see Supplementary Fig. 2).

**Fig. 3:**
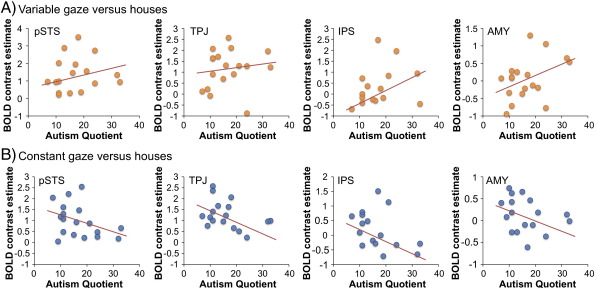
Scatterplots illustrating how AQ scores are associated with viewing BOLD responses to viewing variable (variable gaze versus houses contrast, A) and constant (constant gaze versus houses contrast, B) eye gaze direction in selected regionss in the right hemisphere. BOLD contrast estimates were extracted from coordinates of the peak voxels identified in the AQ by variable versus constant gaze contrast (Table 1). pSTS = posterior superior temporal sulcus, TPJ = temporo-parietal junction, IPS = intraparietal sulcus and AMY = amygdala. Scatterplots are for visualization only and these data were not subject to statistical analysis.

Finally, it is possible that the correlation between AQ scores and activation to variable versus constant gaze was due to an increased response to variable gaze, a decreased response to constant gaze, or both. To explore this further, we examined the correlation between AQ and i) responses to variable gaze vs. house stimuli and ii) constant gaze vs. house stimuli. This demonstrated that for the same regions as shown in Fig. 2, AQ correlated *positively* with variable gaze versus houses and *negatively* with constant gaze versus houses (Fig. 3).

## Discussion

**Fig. 4:**
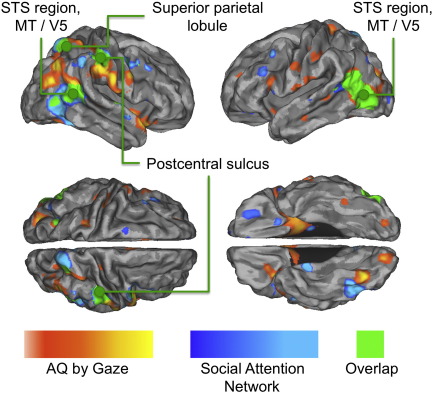
Brain regions showing positive correlations with AQ and activation to variable versus constant gaze direction (red to yellow) and those constituting the brain network for gaze perception (blue to turquoise, adapted from Nummenmaa and Calder, 2009). Green shows areas of overlap.

Our study shows that in typical individuals, the neural response to eye gaze across the social attention network (pSTS, TPJ, amygdala, IPS, SPL, and SMG) is closely related to the number of autism spectrum characteristics they display. The overlap between these AQ-dependent responses and the network for social attention identified by recent meta-analysis (Nummenmaa and Calder, 2009) is illustrated in Fig. 4. Although functional imaging studies have not revealed a specific neural marker of ASD, they have consistently shown abnormal functioning of a number of the same regions that showed AQ-dependent changes in BOLD response in the present study, namely the TPJ, STS and amygdala (see reviews in Baron-Cohen et al., 2000; Di Martino et al., 2009; Zilbovicius et al., 2006). Pelphrey et al. (2005) showed that relative to typical controls, individuals with ASD show less activation to eye gaze cues in areas such as the pSTS. At first sight then, it is surprising that we found a positive association between AQ and the hemodynamic response to variable versus constant gaze. However, our results accord with recent research showing that AQ scores in neurotypical individuals were *positively* related to the change in BOLD response in pSTS produced by a measure of task-independent deactivation (von dem Hagen et al., 2011). The same study also showed that AQ was *negatively* correlated with white matter (WM) volume in a very similar region of pSTS (52, − 42 12) which also falls reasonably close to the region where the AQ-dependent pSTS responses were found in the present study (68, − 42, 24; Euclidian distance between the peaks = 20 mm).

Von dem Hagen et al. (2011) suggested that the enhanced BOLD signal in high AQ participants might reflect a compensatory response for their reduced pSTS WM volume. A similar inverse relationship between WM integrity and increased BOLD response has been seen in the early stages of multiple sclerosis (MS), which is characterised by damage to WM tracts (Rocca et al., 2005; Rocca et al., 2009). Notably, however, after a critical point of white matter deterioration *reduced* BOLD response is found in the later stages of this disease. We do not wish to draw parallels between ASD and degenerative disorders, but rather simply to highlight that the relationship between BOLD response and brain structure is not necessarily positive. It is therefore possible that white matter integrity is negatively correlated with autistic traits throughout the whole AQ range from typical individuals to those with ASD (Barnea-Goraly et al., 2004; von dem Hagen et al., 2011), whereas the BOLD response to social stimuli might show an inverted u-shaped relationship with AQ scores. Hence, increased BOLD response in neurotypical individuals with higher AQ scores could reflect compensatory mechanisms as these individuals may require more cognitive resources to process gaze and other social cues than people with lower AQ scores. However, after some critical point in the AQ distribution (e.g. close to the clinical cut-off) this compensatory processing might fail, at which point, increasing AQ scores begin to show a *negative* association with BOLD responses, resulting in an inverted U-shaped association between AQ and BOLD when the full range of AQ scores are considered. Such an association could reconcile our findings with clinical studies showing decreased BOLD responses to social cues in ASD (Castelli et al., 2002; Pelphrey et al., 2005). In line with this prediction, an inverted-u-shape relationship has already been observed between self-referential cognition and AQ, reflecting a positive association in controls and a negative association in people with ASD (Lombardo et al., 2007). Future research involving both typical and ASD populations should therefore address whether the association between AQ and both the BOLD response and anatomical structure of pSTS across the entire AQ range.

The above explanation is by no means the only possible factor that could underlie the positive relationship between AQ and BOLD response to eye gaze. As far as we are aware, the only previous study showing an abnormal BOLD response to a comparison of two gaze cues in people with ASD used a very different task in which a face directed its gaze towards or away from the position of an object in the display (Pelphrey et al., 2005), depending on the location of the object (i.e., the gaze was the same for both conditions). While Pelphrey and colleagues found reduced pSTS activation in individuals with ASD relative to controls, we cannot discount that people with autism might show increased activation in response to the variable versus constant Gaze conditions used in the current study. In this respect, the important result is that AQ scores were correlated with the activity of a network of regions implicated in gaze processing, rather than the direction of the correlation.

Our study also suggests that the direction of the relationship between BOLD and AQ may be influenced by the nature of the stimuli. Relative to the house baseline, variable gaze showed a positive relationship with AQ, whereas constant gaze showed a negative relationship; indicating that the overall positive correlation was composed of positive and negative associations between AQ and activation to variable and constant gaze conditions, respectively. This may be explained by the relative extent to which processing variable and constant gaze engages the social attention network in low and high AQ participants.

### Autism spectrum traits modulate the activity of the social attention network

Hemodynamic response and AQ scores showed a positive correlation with a number of regions in the social attention network. Next, we deal the potential role of each. The pSTS region has been implicated widely in perception of social stimuli (as reviewed by Allison et al., 2000; Nummenmaa and Calder, 2009), and its involvement in eye gaze perception is well documented (Hoffman and Haxby, 2000; Hooker et al., 2003; Pelphrey et al., 2004b). However, recent studies point to its involvement in analyzing behavioural outcomes and intentionality of human actions (Pelphrey et al., 2004a; Pelphrey et al., 2004b; Saxe et al., 2004). Understanding intentions of others from social cues such as gaze is often impaired in autism (Baron-Cohen et al., 1995), and recent imaging studies have found that when compared to controls, individuals with ASD show an abnormal pSTS response to the perceived intentionality of gaze shifts (Pelphrey et al., 2005) or abstract shapes moving interactively with implied intentionality (such as chasing or teasing, Castelli et al., 2000). The present data from typical individuals accord with these results, as we observed the AQ-correlated pSTS response only for the gaze condition, in which the eyes convey a clear intention of looking at, or being interested in, various locations. All in all, these findings fit well with the proposal that functional changes in the pSTS region may underlie some of the social perception dysfunctions in ASD (Pelphrey et al., 2005). Interestingly, ROI analysis also confirmed that AQ modulated BOLD responses to gaze in the FFA (see supplementary Fig. 1). This accords with findings showing that FFA is involved in processing eye gaze (George et al., 2001; Hooker et al., 2003; Nummenmaa et al., 2010), and is in line with the proposal that the ventral face perception route may also significantly contribute to the processing of changeable facial cues (see reviews in Calder et al., 2011; Calder and Young, 2005)

By contrast, AQ did not correlate with the change in BOLD response in the head condition in which the eyes were closed, thus conveying no intention of attending anywhere. Given that cueing effects from averted gaze are maximal when they are presented in the context of a full-face view of the head (Hietanen, 1999), it is interesting that the correlation was also absent for the eyes-only condition where head direction information was not available. It is therefore possible that the present results reflect the activity of cells coding both head and gaze direction information, and their interactions with other components of the social attention network (Perrett et al., 1992). Alternatively, the eye region alone may constitute a less natural, ‘less social’ stimulus than gaze shown in the context of a fully visible head.

It is also interesting to note that pSTS activation was not observed for the group-level analyses of variable vs. constant gaze or variable vs. constant eyes-only contrasts. Although many studies report pSTS activation in gaze perception tasks (Engell and Haxby, 2007; Hooker et al., 2003), some don't (George et al., 2001; Kawashima et al., 1999). Furthermore, even when effects are observed they tend to be small, suggesting variable evidence exists across subjects. On the basis of the present results showing AQ-dependent pSTS responses to gaze, AQ score might be a factor in determining whether or not the eye gaze perception systems respond to gaze stimuli. In other words, it is possible that the variability in AQ scores in the typical population may account for discrepant findings in the gaze perception literature. For example, relative increases and decreases in pSTS activation in participants scoring high and low on the AQ questionnaire respectively, as shown here, may result in no overall mean change in BOLD response in this region. In this respect, it is worth emphasising that the main effect of an experimental manipulation and its correlations with proxy variables, such as personality scores, measure different things and are statistically distinct. Main effects can occur in the absence of correlations, and vice versa (see review in Calder et al., 2011).

Although TPJ is not typically associated with gaze perception, it has been consistently linked with mentalizing or theory of mind processing (Decety and Lamm, 2007; Saxe and Kanwisher, 2003; Saxe and Wexler, 2005), and with directing attention to behaviourally salient events (Corbetta and Shulman, 2002). In the context of the present study, it seems plausible that the association between AQ scores and TPJ activation might reflect individual differences in the spontaneous tendency to draw mentalistic inferences from eyes, given that spatial reorienting (as indexed by the Posner cueing task) is typically intact in autism (Iarocci and Burack, 2004). AQ was also correlated with activation in the vicinity of the motion-sensitive MT/V5 complex which has been proposed to constitute an initial stage in processing dynamic facial characteristics, including gaze shifts (Nummenmaa et al., 2010; O'Toole et al., 2002). A magnetoencephalographic study has also shown that MT/V5 may extract social information conveyed by the eyes within 160 ms post-stimulus, as it shows stronger responses to faces that establish gaze contact rather than gaze aversion (Watanabe et al., 2006). The AQ-dependent MT/V5 activation shows that autistic traits could influence even the very early, dynamic face processing stages. This raises the possibility that facial processing deficits observed in ASD (see Golarai et al., 2006) could be partially accounted for by deficits in extracting motion cues from faces, although this would need to be verified with studies of individuals with ASD.

Several studies suggest that the amygdala's role in gaze perception relates to drawing mentalistic inferences from eyes (Baron-Cohen et al., 1999) and detecting or monitoring gaze contact (Hooker et al., 2003; Kawashima et al., 1999), but its exact contribution is currently unclear. ASD does not typically impair discrimination of others' gaze direction, but hampers the ability to infer others' mental states (e.g., intentions) from their gaze (Baron-Cohen et al., 1995; Nation and Penny, 2008). Taking this dissociation into account, the AQ-dependent amygdala activation found in the current study seems likely to reflect the amygdala's role in attaching social or affective salience to perceived gaze direction, rather than gaze direction encoding per se. The amygdala correlation with AQ is also consistent with Baron-Cohen's proposal that amygdala dysfunction could be one of the potential precursors to ASD (Baron-Cohen et al., 2000).

Previous research has shown that circuits involved in visual attention are also engaged by viewing changes in gaze direction. The IPS forms part of the dorsal attention system which is thought to underlie attentional target selection (Corbetta et al., 2008). Haxby et al. (2000) proposed that the IPS could also subserve attention orienting from seen gaze direction. In line with this proposal, single-unit recordings in macaques (Shepherd et al., 2009) have indeed established that some neurons in the lateral intraparietal area (LIP; the lateral wall of the monkey IPS) increase their firing rate, while others reduce their firing rate, when the monkey views an image of a conspecific gazing towards the cell's response field. Similar effects are also observed when the monkey overtly looks at the corresponding locations, suggesting this regions' involvement in mirroring others' gaze. A number of behavioural studies in humans have found that viewing averted gaze triggers an involuntary shift of covert or overt attention towards the gazed-at location (for a review see Frischen et al., 2007). Although attention orienting was not measured behaviourally in our study, it is plausible that the AQ-dependent response in the IPS reflects the relationship between AQ and the tendency to imitate others' gaze behaviour (Bayliss and Tipper, 2005). In addition to the IPS, AQ-dependent activation of the IPL and SMG was also observed. The role of these regions in governing goal-directed shifts of attention is well established (Corbetta et al., 2008; Corbetta and Shulman, 2002), and a recent meta-analysis of fMRI studies on autism (Di Martino et al., 2009) showed that individuals with ASD show reduced IPL and SMG activations in non-social attentional tasks.

Finally, it must be noted that although there was a considerable overlap between the social attention network (Nummenmaa and Calder, 2009) and AQ-modulated BOLD responses to gaze in the present study (Fig. 4), some of the activation foci clearly fall outside the social attention network. It is therefore possible that AQ also influences certain ‘gaze-independent’ neural mechanisms. However, this seems unlikely, given that AQ was not correlated with responses to variable versus constant heads and eyes-only conditions. Perhaps a more likely explanation is that AQ modulated responses in brain regions that are not part of the ‘core’ network for social attention perception, but are nevertheless recruited during certain gaze perception tasks such as the one used in the present study.

## Conclusions

We have shown that individual differences in autism spectrum traits in typical individuals are correlated with brain responses to observed shifts in gaze direction in key components of the brain circuit for eye gaze perception (Nummenmaa and Calder, 2009), as well as those involved in inferring others' mental states (Saxe and Kanwisher, 2003; Saxe and Wexler, 2005). Dysfunction in these same regions is also systematically observed in individuals with ASD. Our results therefore provide support for the existence of a broader autistic spectrum that extends into the typical population (Baron-Cohen, 1995; Baron-Cohen et al., 2001b; Frith, 1991). The data also demonstrate that neural processing of eye gaze is not fully homogenous across typical participants, as individual differences in social-cognitive processing styles indexed by AQ have a profound influence on the neural processing of eye gaze. Thus, in addition to furthering our understanding of the neurobiological basis of the extended autism phenotype, our results demonstrate that studies of eye gaze perception in typical individuals should take into account that a significant proportion of between-subject variance has a meaningful psychological basis. The identification of a cortical network influenced by AQ may be important in furthering our understanding of the neurobiological basis of deficits in the processing of social cues. Future studies on the development and functional connectivity of this network across the entire autism spectrum range could contribute to our understanding of neural markers of the development of ASD.

## Figures and Tables

**Table 1:** Brain regions showing positive correlations (*p* < 0.05 FDR corrected) between AQ and activation to variable versus constant eye gaze direction.

Cerebral region	Laterality	X	Y	Z	T
Middle occipital gyrus, MT/V5	L	− 42	− 66	2	7.87
Middle occipital gyrus, Angular gyrus	R	38	− 72	22	5.97
Supramarginal gyrus	R	50	− 26	38	6.15
Superior temporal sulcus	R	68	− 42	24	5.89
Superior temporal sulcus	R	50	− 50	18	4.38
Superior temporal gyrus	L	− 66	− 24	12	3.59
Superior temporal gyrus	R	52	− 28	6	3.88
Pallidum, Putamen	L	− 14	− 10	− 8	5.29
Fusiform gyrus (FFA)	R	30	− 68	− 20	4.98
Superior frontal gyrus	L	− 18	36	24	4.85
Intraparietal sulcus	R	34	− 48	60	4.43
Insula	L	− 44	12	− 8	3.85
Insula	R	26	24	8	4.43
Amygdala	L	− 26	− 8	− 12	4.37
Amygdala	R	26	− 4	− 14	3.81
Supramarginal gyrus/ Inferior pariatal lobule	L	− 42	− 30	36	4.35
Middle and anterior cingulate	R	4	12	26	4.22
Middle cingulate	R	18	− 28	52	4.02
Frontal operculum/ Superior temporal gyrus	L	− 48	− 16	16	4.01
Frontal operculum	L	− 32	8	22	3.97
Temporo-parietal junction	R	52	− 58	8	3.90
Lingual gyrus	L	− 20	− 88	− 14	3.60
Lingual gyrus	R	16	− 92	− 12	3.88
Rolandic operculum	L	− 46	14	14	3.85
Central sulcus	L	− 22	− 44	60	3.77
Middle temporal gyrus	L	− 64	− 14	− 18	3.57
