# Supplementary material for: Autism spectrum traits predict the neural response to eye gaze in typical individuals
Source: Neuroimage. 2012 Feb 15;59(4-4):3356–63. doi: 10.1016/j.neuroimage.2011.10.075 (PMC3315678; doi:10.1016/j.neuroimage.2011.10.075)
Supplement: Supplementary file 1 — Supplementary material. [file mmc1.doc]

Supplementary material

**1. Region of Interest Analysis**

In order to test whether AQ would modulate brain responses to gaze specifically in areas associated with face and gaze perception (fusiform face area, FFA; occipital face area, OFA; pSTS, intraparietal sulcus, IPS; amygdala) and mentalizing (temporo-parietal junction; TPJ), we defined right-hemispheric regions of interest (ROIs) using data from prior, independent studies. For FFA, OFA and pSTS, we defined 8-mm spheres using as centers the local maxima from a study assessing the brain networks for face perception (FFA: 48, -57, -33; OFA: 54, -72, 15; pSTS: 48, -57, -33; Pourtois et al., 2005). For IPS the coordinates were derived from a previous study assessing the role of IPS across various attentional tasks (32, –47, 56; Wojciulik and Kanwisher, 1999) and for TPJ from a study assessing the role of TPJ in mentalizing (52, -52, 23; averaged across two experiments in Saxe and Kanwisher, 2003). The amygdala ROI was defined using the WFU pick atlas (Maldjian et al., 2003) and AAL (Tzourio-Mazoyer et al., 2002) atlas. Subsequently, ROI analysis with small-volume-corrections (SVCs) and a statistical threshold of *p* < .05 FWE corrected was used to assess the association between AQ and response to variable versus constant eye gaze in these ROIs. This analysis confirmed that AQ was associated with response to variable versus constant eye gaze in all the tested ROIs, p < .05 SVC. (see Supplementary Figure 1).


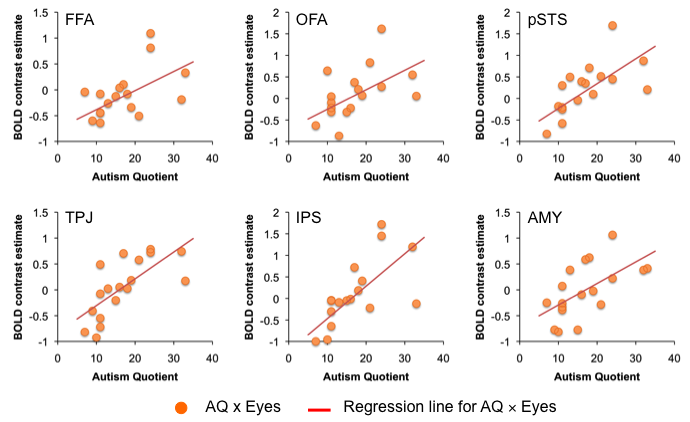


**Supplementary Figure 1** Scatterplots visualizing the association between AQ and averaged BOLD contrast responses to variable versus constant eye gaze in the *a priori* regions of interest. FFA = fusiform face area, OFA = occipital face area, pSTS = posterior superior temporal sulcus, TPJ = temporo-parietal junction, IPS = intraparietal sulcus, AMY = amygdala.

**2. Analysis of The Subscales of The AQ**

**
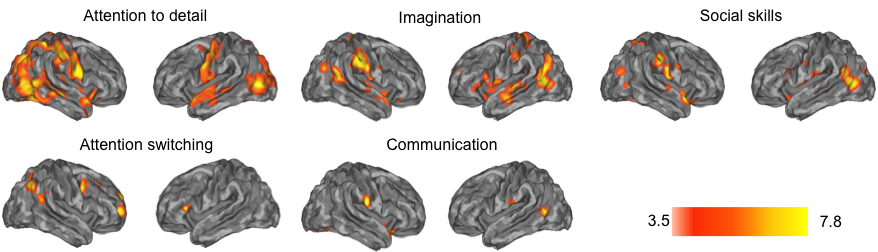
**

**Supplementary Figure 2** Brain regions showing positive correlations between AQ subscale scores and activation to variable versus constant eye gaze direction (*p* < .005, uncorrected). Colour bar denotes *t*-statistic range.

References

Maldjian, J.A., Laurienti, P.J., Kraft, R.A., Burdette, J.H., 2003. An automated method for neuroanatomic and cytoarchitectonic atlas-based interrogation of fMRI data sets. Neuroimage 19, 1233-1239.

Pourtois, G., Schwartz, S., Mohamed, L., Seghier, M.L., Lazeyras, F., Vuilleumier, V., 2005. Portraits or people? Distinct representations of face identity in the human visual cortex. Journal of Cognitive Neuroscience 17, 1043–1057.

Saxe, R., Kanwisher, N., 2003. People thinking about thinking people: The role of the temporo-parietal junction in "theory of mind". Neuroimage 19, 1835–1842.

Tzourio-Mazoyer, N., Landeau, B., Papathanassiou, D., Crivello, F., Etard, O., Delcroix, N., Mazoyer, B., Joliot, M., 2002. Automatic anatomical labelling of activations in SPM using a macroscopic anatomical parcellation of the MNI MRI single-subject brain. Neuroimage 15, 273-289.

Wojciulik, E., Kanwisher, N., 1999. The generality of parietal involvement in visual attention. Neuron 23, 747-764.
